# How necessary and feasible are reductions of methane emissions from livestock to support stringent temperature goals?

**DOI:** 10.1098/rsta.2020.0452

**Published:** 2021-11-15

**Authors:** Andy Reisinger, Harry Clark, Annette L. Cowie, Jeremy Emmet-Booth, Carlos Gonzalez Fischer, Mario Herrero, Mark Howden, Sinead Leahy

**Affiliations:** ^1^ Australian National University, Canberra, Australia; ^2^ New Zealand Agricultural Greenhouse Gas Research Centre (NZAGRC), Palmerston North, New Zealand; ^3^ New South Wales Department of Primary Industries/University of New England, Armidale, Australia; ^4^ Department of Global Development, College of Agriculture and Life Sciences, and Cornell Atkinson Centre for Sustainability, Cornell University, Ithaca, USA

**Keywords:** agriculture, methane, marginal warming, land use, sequestration, Paris Agreement

## Abstract

Agriculture is the largest single source of global anthropogenic methane (CH_4_) emissions, with ruminants the dominant contributor. Livestock CH_4_ emissions are projected to grow another 30% by 2050 under current policies, yet few countries have set targets or are implementing policies to reduce emissions in absolute terms. The reason for this limited ambition may be linked not only to the underpinning role of livestock for nutrition and livelihoods in many countries but also diverging perspectives on the importance of mitigating these emissions, given the short atmospheric lifetime of CH_4_. Here, we show that in mitigation pathways that limit warming to 1.5°C, which include cost-effective reductions from all emission sources, the contribution of future livestock CH_4_ emissions to global warming in 2050 is about one-third of that from future net carbon dioxide emissions. Future livestock CH_4_ emissions, therefore, significantly constrain the remaining carbon budget and the ability to meet stringent temperature limits. We review options to address livestock CH_4_ emissions through more efficient production, technological advances and demand-side changes, and their interactions with land-based carbon sequestration. We conclude that bringing livestock into mainstream mitigation policies, while recognizing their unique social, cultural and economic roles, would make an important contribution towards reaching the temperature goal of the Paris Agreement and is vital for a limit of 1.5°C.

This article is part of a discussion meeting issue 'Rising methane: is warming feeding warming? (part 1)'.

## 1. Introduction

Agriculture, including associated emissions from deforestation, accounts for about 21% of total annual anthropogenic greenhouse gas emissions when emissions are weighted using the Global Warming Potential with a time horizon of 100 years (GWP_100_; [1]). Agriculture is the largest single source of global methane (CH_4_) emissions from human activities. About 80% of agricultural CH_4_ arises from livestock systems, of which almost 90% comes from enteric fermentation by ruminants such as cattle and sheep, and about 10% from animal manure [2]. The remaining 20% arise primarily from rice paddies with a minor contribution from agricultural residue burning. Global livestock CH_4_ emissions in 2017 were estimated to be around 115 Mt CH_4_, an increase of 10–13% relative to the average in 2000–2006 [3,4].

Agricultural CH_4_ emissions are projected to increase by about 30% in 2050 relative to 2010 under current policies (FAOSTAT; [5]), with a range from 20 to 50% in integrated assessment models (IAMs; [6–8]). Increases are due to a growing human population and increasing demand for animal protein as incomes rise, but with significant variations in demands and trends between regions and countries [7,9,10].

Limiting warming to 1.5°C, the ambitious end of the temperature goal of the Paris Agreement,^1^ would require opposite emission trends. Global agricultural CH_4_ emissions reduce by 24–47% (interquartile range) and carbon dioxide (CO_2_) emissions reach net-zero by mid-century in modelled pathways that limit warming to 1.5°C with no or limited overshoot at least global cost [7]. More than 100 countries include agriculture in their nationally determined contributions (NDCs; [11]). However, most NDCs lack details and few (including industrialised) countries have specific targets or are designing policies that could drive absolute sector-wide reductions of agricultural CH_4_ emissions [12,13]. By contrast, at least 46 countries had price-based policies that target CO_2_ emissions from fossil fuels implemented or scheduled for implementation in 2020 [14].

This study does not aim to explore systematically why the ambition of agricultural mitigation policies has remained limited so far. Key factors are likely to include the small share of agricultural emissions in national totals for most industrialized countries, high levels of economic protection for agricultural producers in many countries, and the critical role of agriculture to achieve nutrition goals, rural development and poverty alleviation in many developing countries [9,15,16].

The development of more ambitious agricultural climate policies could further be hampered by the perception that CH_4_ is fundamentally less important than CO_2_ as a target for mitigation. Given that CO_2_ persists in the atmosphere over centuries to millennia and hence accumulates over time [17,18], net CO_2_ emissions must drop to zero for temperature to stabilize, and additional warming will occur until that condition is reached. By contrast, CH_4_ has an atmospheric lifetime of approximately 12 years and emissions do not accumulate over centuries; hence, even a very moderate reduction of global CH_4_ emissions at a rate of about 0.3% per year would stabilize warming from CH_4_ at approximately current levels [19–21].

These different temperature outcomes have led some authors to argue that expressing CH_4_ emissions as ‘CO_2_-equivalent’ emissions based on the common 100-year Global Warming Potential (GWP_100_) is misleading and dangerous as it could misdirect attention from the need to reduce global net CO_2_ emissions to zero as quickly as possible [22–25].

These concerns have led some to maintain that deep reductions of agricultural CH_4_ emissions are not necessary to support ambitious climate action (e.g. [26–29]). This view is further supported by an interpretation that slowly declining CH_4_ emissions already represent climate neutrality, given that this would not result in additional warming compared to the present (e.g. [30]), and more rapid reductions would effectively give a free ride to CO_2_ emitters [31]. The biological origin of livestock CH_4_ emissions can further add to the perception that these emissions are part of a natural cycle and hence fundamentally less problematic than the burning of fossil fuels (e.g. [27]).

Differing perspectives on the importance of livestock CH_4_ are the focus of continued debate in New Zealand, the only country currently planning to implement a comprehensive price-based policy to reduce agricultural CH_4_ emissions [32]. Similar discussions may arise in other countries where reductions in agricultural emissions will become increasingly necessary to achieve ambitious long-term economy-wide emission reduction targets. For example, the UK and all states of Australia have adopted targets for net-zero emission of all greenhouse gases including methane for 2050; modelled pathways to achieve such goals include carbon dioxide removals along with substantial reductions in livestock CH_4_ emissions, but the scale of both carbon dioxide removals and of livestock CH_4_ reductions are politically contested [33–36].

Given this context, the purpose of this study is twofold: one is to clarify the extent to which global reductions in CH_4_ emissions from livestock are necessary to support the temperature goal of the Paris Agreement (§2), touching also on whether such reductions might be seen as fair and consistent with the way CO_2_ emissions are treated. The second purpose is to consider the feasibility of such CH_4_ reductions, focusing particularly on the potential of novel technologies to increase the mitigation potential in some production systems (§3). We then move beyond a narrow focus on CH_4_ to consider the role of livestock in landscape-based solutions to climate change (§4). Our conclusions summarize key insights for the development of policies consistent with the goals of the Paris Agreement.

## 2. How much are livestock contributing to climate change?

Enteric fermentation and manure management together contribute roughly 30% of total anthropogenic CH_4_ emissions [3]. The radiative forcing from anthropogenic CH_4_ emissions, including indirect effects, has been estimated at just over 40% of total radiative forcing from all human activities in 2011 [17], which approximates its share in anthropogenic warming. This suggests that CH_4_ emissions from livestock are responsible for roughly 12% of anthropogenic warming to date. Detailed model simulations give a similar magnitude, at 14% [37]. The precise contribution is subject to updated estimates of radiative efficacy (e.g. [38]) and tropospheric ozone chemistry, which constitutes a key indirect warming from CH_4_ emissions [39–41].

The significant contribution from livestock CH_4_ emissions to current warming does not, however, determine how much those emissions must be reduced to support the temperature goal of the Paris Agreement. While the need to reduce the dominant, long-lived greenhouse gas CO_2_ to zero is unambiguous, the same does not apply to CH_4_, owing to the different lifetimes of these gases and temperature response to their emissions.

**Figure 1.**
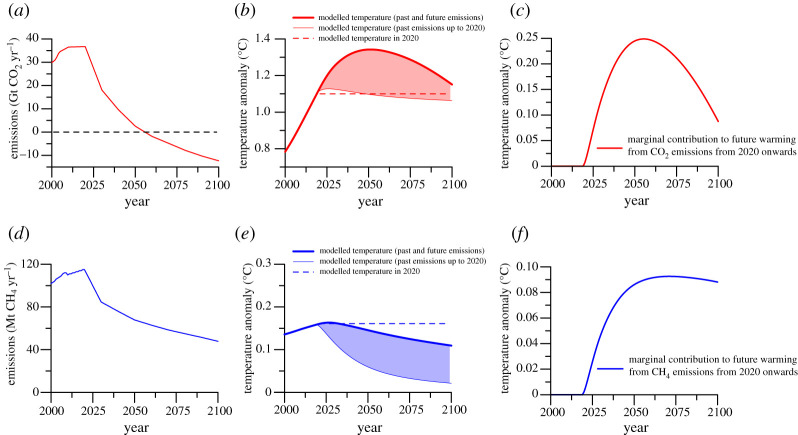
Temperature change associated with global net carbon dioxide (*a*–*c*) and livestock methane (*d*–*f*) emissions. (*a*,*d*) Global emissions for pathways consistent with limiting warming to 1.5°C with no or limited overshoot, using the average of five RCP1.9-SSP1 scenarios from the IPCC SR15 database [42]. (*b*,*e*) Temperature change due to historical and projected future emissions (thick solid lines) and historical emissions up to 2020 only (thin solid lines). The shaded areas indicate the contribution to warming from future emissions. (*c*,*f*) Temperature change due to future emissions only. Data sources: emissions scenarios are from the scenario database of the IPCC Special Report on Global Warming of 1.5°C [42,43]. Livestock CH_4_ emissions are assumed to be a constant fraction of AFOLU (Agriculture, Forestry and Other Land Use) CH_4_ emissions in these pathways. Temperature has been modelled using a simplified pulse response model based on simulations using MAGICC [44]. (Online version in colour.)

Figure 1 illustrates these differences by showing the global net CO_2_ and livestock CH_4_ emissions and associated temperature change in pathways that limit overall warming to 1.5°C above pre-industrial levels. Emissions reflect the average of five IAMs based on the SSP1 (global sustainability) socio-economic scenario as assessed in Rogelj *et al*. [7]. In these pathways, CO_2_ emissions reach net-zero by about 2055 and become negative thereafter, while livestock CH_4_ emissions drop 38% by 2050 relative to 2010 and decline further to 2100. These pathways result in just over 1.5°C peak warming around 2050 followed by a gradual decline.

The contributions from CO_2_ and CH_4_ to overall warming differ sharply in these pathways (figure 1*b*,*e*). Warming from CO_2_ emissions continues to increase until emissions reach net-zero and declines only once CO_2_ is actively removed from the atmosphere (through a combination of large-scale use of bioenergy with carbon capture and storage (BECCS) and afforestation). Even so, warming from CO_2_ is higher in 2100 than in 2020. By contrast, warming from livestock CH_4_ declines below current levels as soon as emissions drop significantly. Even though CH_4_ emissions remain well above zero, the overall warming from livestock CH_4_ in these pathways in 2100 is below the warming they caused in 2020.

These different behaviours have led some researchers to argue that using GWP_100_ is not just inaccurate but gives the wrong sign for the change in temperature if CH_4_ emissions are declining [19,23]. Going further, given this mismatch between cumulative emissions and temperature change, it has been suggested that the use of GWP to inform climate change mitigation strategies would be unfair [45], as even rapidly-reducing CO_2_ emissions would cause additional warming, whereas rapid reductions in livestock CH_4_ emissions would cause less warming in 2050 than today; yet CH_4_ emitters would be under continued pressure to reduce their remaining emissions even further.

We suggest that care needs to be taken to disentangle the effect of past and future emissions on the future climate, and in assigning responsibility for temperature changes related to ongoing emissions.

Figure 1 shows (middle and right-hand panels) that even though rapidly declining livestock CH_4_ emissions result in less warming in future than today, the climate in 2050 will be substantially warmer with, compared to without, these future livestock CH_4_ emissions. In other words, future CH_4_ emissions make a substantial *marginal* contribution to climate change (borrowing from the meaning of this term in economics, we use ‘marginal warming’ to denote the warming that an additional emission causes, relative to the absence of that emission, all else being equal). CH_4_ emissions are no different in that regard to CO_2_. By the end of the twenty-first century, the marginal warming from livestock CH_4_ emissions in these deep mitigation pathways is almost 0.1°C, more than one-third of the marginal warming from global net CO_2_ emissions of 0.25°C in 2050 (i.e. the warming from future net CO_2_ emissions, compared to the absence of those emissions). By 2100, the marginal warming from CO_2_ would drop below 0.1°C, given the large-scale net removal of CO_2_ envisaged in these pathways, and thus reach a similar level as the marginal warming from livestock CH_4_ emissions.

As decisions available today can only control future activities, efficient mitigation strategies must consider marginal contributions of different sectors and gases to future temperature, as this provides a measure of how much warming could be avoided by reducing future emissions or enhancing removals. Consideration of marginal warming makes it clear that future livestock CH_4_ emissions will be a significant contributor to future warming, not cooling: each future tonne of CH_4_ emitted will make the climate warmer than it would be otherwise, regardless of whether emissions are rising or declining over time. This is also reflected in the social cost of methane, which is uncertain but substantially greater than the social cost of carbon on a tonne for tonne basis [46–48]. Additional non-climate damages from CH_4_ emissions arise from increased tropospheric ozone that can negatively affect crop production and human health [49]. Efforts to reduce CH_4_ emissions as much as socially, environmentally and economically possible via policies that place the cost of CH_4_ emission reductions on CH_4_ emitters would, therefore, be conceptually fully consistent with a ‘polluter pays’ principle.

Nonetheless, various policy choices are possible on how to share the cost of emission reductions across society (e.g. instead of ‘polluter pays’, one could adopt a ‘beneficiary pays’ principle), and what scale of reductions is socially and economically feasible and acceptable. However, these choices are relevant for all greenhouse gases and emitters; CH_4_ from livestock does not occupy a special role in this regard simply because of its short lifetime or biogenic origin.

There are also valid questions on how to recognize the contribution from past CO_2_ emissions and other long-lived gases to future global warming in the design of climate policies. However, this raises fundamentally different ethical and policy questions compared to those dealing with the warming caused by future emissions. Future CO_2_ emitters are not necessarily the same as those responsible for past CO_2_ emission, and the benefits of past CO_2_-emitting activities tend to be spread across today's society (particularly in the case of past deforestation that provides the land for today's livestock agriculture). How countries might incorporate the warming from past CO_2_ emissions into climate policy thus raises questions that go well beyond the scope of this study. Nonetheless, we argue that a clear separation of legacy warming from past emissions (which is significant only for long-lived gases), and marginal warming from current and future emissions and removals (which applies for all gases) may support a more constructive conversation about how to best address emissions of different gases in mitigation strategies.

**Figure 2.**
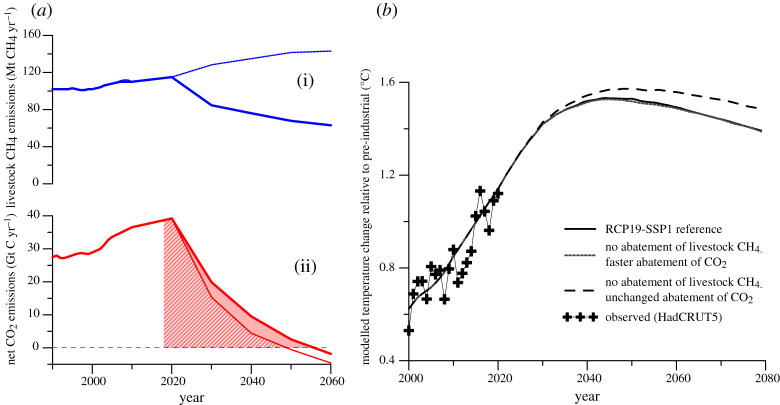
Emission pathways of livestock CH_4_ (*a*(i)) and net CO_2_ (*a*(ii)) consistent with limiting warming to 1.5° with no or limited overshoot, and modelled temperature outcomes (*b*). The default emission pathways (solid lines) are the same as in figure 1. The dotted lines are for a business-as-usual scenario with a roughly 30% increase in livestock CH_4_ emissions by 2050 (relative to 2010), and net CO_2_ emissions adjusted such that the modelled temperature change remains virtually identical. Shaded and hashed areas in *a*(ii) indicate the remaining carbon budget for those pathways (starting in 2018; for details, see text). (*b*) Temperature responses, including for a scenario where livestock CH_4_ emissions are not reduced and emissions from all other sectors remain unchanged (dashed line). Temperature has been modelled using MAGICC 6.3 [44], using the median of a probabilistic set of 600 runs. Observed temperature change to 2020 is shown for illustration (crosses), using 1986–2005 as reference level, using HadCRUT5 [50]. (Online version in colour.)

Choices about abatement of livestock CH_4_ emissions strongly influence the amount of CO_2_ that can be emitted while remaining within stringent temperature limits. Figure 2 illustrates this trade-off by comparing global net CO_2_ and livestock CH_4_ emissions in 1.5°C-consistent pathways with a hypothetical alternative scenario where livestock CH_4_ emissions increase consistent with current policies rather than decrease, and CO_2_ emissions are adjusted downwards to achieve the same overall temperature outcome.

If livestock CH_4_ emissions rise rather than fall, as shown in figure 2, we find that this would require a significantly more rapid reduction of net CO_2_ emissions between 2020 and 2030 (by 6.3% per annum rather than 4.9%), reaching net-zero emissions almost a decade earlier (by 2048 rather than 2055), and more and earlier net CO_2_ removal. The pace and scale of CO_2_ reductions to limit warming to 1.5°C are already highly ambitious and at the limit of feasibility [43], and even tighter constraints make this limit even less feasible. Of course, the world is not yet on track for emission reductions consistent with 1.5°C or even ‘well below’ 2°C [51], but this analysis shows that excluding livestock CH_4_ emissions from transformative changes could jeopardize the 1.5°C limit even if all other sectors undertook the rapid emission reductions necessary to reach global net-zero CO_2_ emissions in the 2050s [52].

The trade-off between reducing livestock CH_4_ emissions and fossil CO_2_ emissions (or other greenhouse gases) can also be quantified via the ‘remaining carbon budget’. This is defined as the cumulative amount of CO_2_ emissions, up to net-zero, that would be consistent with limiting warming to a specified level while considering the contribution of non-CO_2_ climate forcers to total warming.

The remaining carbon budget consistent with a 50/50 chance of limiting warming to 1.5°C has been estimated to be about 580 Gt CO_2_ from 2018, but this already assumes rapid concurrent reductions of non-CO_2_ emissions as in figure 1 [7]. If, instead, livestock CH_4_ emissions increase as in figure 2, we find that the cumulative CO_2_ emissions consistent with the same temperature outcome are reduced by about 136 Gt CO_2_, or almost one-quarter from 580 to 444 Gt CO_2_. For a temperature limit of 1.5°C, livestock CH_4_ emissions, therefore, act as a highly sensitive lever on the remaining carbon budget. The relative influence would be smaller but remains significant for less ambitious temperature goals. For example, the remaining carbon budget to limit warming to 2°C with 66% probability (often interpreted as warming remaining ‘well below’ 2°C) is estimated at 1170 Gt CO_2_ [7], and failure to mitigate livestock CH_4_ emissions would reduce this budget by about 12%.

The difference between mitigated and unmitigated livestock CH_4_ emissions amounts to a temperature difference of about 0.1°C towards the end of the twenty-first century (figure 2). Failure to reduce livestock CH_4_ emissions would, therefore, not in itself cause warming to exceed 2°C. Whether mitigation of livestock CH_4_ emissions is viewed as necessary to achieve the temperature goal of the Paris Agreement, thus depends on how concerned we are about adding 0.1°C to the warming caused by all other sectors and gases. However, this concern is not unique to livestock CH_4_, since every individual sector makes only a small contribution to global warming; yet, if every sector were to avoid mitigating its emissions because of this, warming would almost certainly exceed 2°C [43].

We emphasize that these conclusions hold irrespective of greenhouse gas emissions metrics and despite the short lifetime of CH_4_ in the atmosphere. A focus on marginal warming demonstrates that future livestock CH_4_ emissions make a significant, positive contribution to future warming. This contribution is more than one-third of the contribution of future CO_2_ emissions to peak warming around 2050 in the most stringent mitigation pathways.

The pressure of livestock production on the remaining carbon budget is even more pronounced if both CH_4_ and nitrous oxide (N_2_O) emissions from livestock are considered and remain unabated [12], or if the food system as a whole is considered [53]. The trade-off between mitigating livestock emissions and CO_2_ from other sectors, along with the need for land-based CO_2_ removal to remain within a tight remaining carbon budget, points to the need to adopt a broader landscape approach to evaluate effective mitigation strategies. We return to this integrated perspective in a later section, but first, consider the prospects for reducing livestock CH_4_ emissions at the global scale.

## 3. Prospects for livestock methane mitigation in the near and long term

Reductions of livestock CH_4_ emissions can occur via supply and demand-side approaches. In this section, we briefly summarize existing supply-side options but do not attempt a detailed review (for recent comprehensive reviews, see [9,54–56]) and then focus on the potential of emerging technologies. The complementary role of demand-side approaches is considered in the next section.

Supply-side interventions can be grouped into the use of different feeds and feed additives, measures to increase feed quality, increased livestock and crop/pasture productivity (e.g. increased growth rates, milk yields and animal fertility), and manure management through aeration or biogas production and use [55]. The mitigation potential from these measures varies across studies, with the technical potential to reduce CH_4_ emissions from enteric fermentation estimated at up to about 50 Mt CH_4_ yr^−1^ by 2050 [54], and up to about 5 Mt CH_4_ yr^−1^ from manure management. The economic potential is generally smaller, with reductions of less than 20 Mt CH_4_ yr^−1^ at carbon prices of up to US$100/tCO_2_-eq [9,56]. For reference, livestock CH_4_ emissions are projected to rise to more than 140 Mt CH_4_ under current policies by 2050, whereas modelled emissions in 1.5°C-consistent pathways fall to about 70 Mt CH_4_ [7].

In the absence of targeted mitigation policies, currently feasible practices are those that deliver mitigation as a co-benefit to improved production efficiency [57]. These approaches primarily reduce emissions intensity, which can serve as an important entry point for mitigation efforts in developing countries, given the co-benefits of productivity gains for rural development and food security [58–61]. However, enhanced production efficiency on its own may offset environmental benefits through increased resource use and increasing economic incentives to expand into marginal lands. Reducing absolute emissions relies on complementing reductions in emissions intensity with measures to limit overall demand and/or land use [12,62–65].

Overall, existing assessments of supply-side mitigation options indicate that it will be immensely challenging to achieve the abatement indicated in cost-effective mitigation pathways, especially given the limited development of dedicated policies so far [12,13]. However, such assessments typically do not consider emerging supply-side technologies either in sectoral bottom-up or IAM-based top-down mitigation scenarios. We suggest that novel technologies could achieve significantly stronger supply-side reductions of CH_4_ emissions in some livestock systems and/or make reductions more feasible. Relevant technologies include novel feeds such as genetically modified ryegrass, physical CH_4_ capture and neutralization devices, and feed additives including CH_4_ inhibitors, as well as vaccines.

In the remainder of this section, we focus on four areas that are closest to commercialization and/or are under active development, and that could materially change global emissions, namely synthetic CH_4_ inhibitors; a CH_4_ vaccine; low-emissions breeding and the use of seaweed as a feed additive.

### (a) Methane inhibitors: synthetic

A CH_4_ inhibitor is a chemical compound that suppresses the activity of CH_4_ forming microbes (methanogens) in the rumen. Inhibitors could be delivered as a feed additive or as a bolus (a small capsule containing the active compound, inserted into the rumen). 3-Nitrooxypropanol (3-NOP) has been shown to consistently reduce CH_4_ emissions by around 30% in Total Mixed Ration (TMR) farm systems [66,67] without compromising animal productivity [68] and is expected to be commercially available in some countries within the next 2 years. 3-NOP has limited applicability in grazing systems as it decays within a few hours in the rumen, but its applicability could be extended to most dairy systems via slow-release formulations [69,70]. Research is also progressing into the use of 3-NOP in young ruminants to stimulate lifetime reductions [71], and other inhibitors with longer rumen lifetimes and low dosage rates to allow bolus delivery [72,73].

These developments could increase the utility of CH_4_ inhibitors beyond TMR systems into grazing systems of moderate to high management intensity. In the absence of significant co-benefits for animal performance, adoption of CH_4_ inhibitors will depend on cost and, therefore, climate policy incentives or consumer demand.

### (b) Methane vaccine

Vaccination against the rumen methanogens is expected to have broad applicability globally [74] and could be practical and cost-effective even in extensive systems. Research into a CH_4_ vaccine remains in the development phase and has not yet been demonstrated in live animals. However, all major components of a vaccine chain have been demonstrated: genome sequencing of methanogens has identified targets that stimulate antibody production; antibodies can be created by host animals and detected in saliva and the rumen; and those antibodies have been shown to suppress pure methanogen cultures *in vitro* [75–77]. The efficacy of a vaccine is necessarily speculative, but a reduction of 30% is considered plausible, given the efficacy of CH_4_ inhibitors.

Commercial availability of a vaccine is estimated to take 7–10 years after demonstration of a prototype. Vaccine adoption could be facilitated by administering it in combination with other widely used animal vaccines. However, adoption rates will depend not only on costs but also on veterinary practices, as many animal vaccines are not adopted fully even where proven to be cost-effective [78].

### (c) Breeding low-emission animals

Sheep vary naturally in the amount of CH_4_ they produce per kilogram of dry matter consumed. This trait has been shown to be heritable and thus enables the breeding of low-CH_4_ emitting sheep [79]. Emissions differ by approximately 10% after three generations, without adverse effects on major production traits and with some positive correlations [80]. Following industry trials, the low-CH_4_ trait is expected to be available to sheep farmers in New Zealand within the next 1–2 years [81,82]. Cattle show similar potential for breeding strategies [83–85], but commercialization is less advanced due to the higher cost of measuring low-emitting animals. Research is underway to develop proxy indicators (e.g. based on milk constituents, rumen microbial profiles) to enable cheap and rapid identification of low-emitting animals [86,87].

Adoption of breeding approaches is subject to breeding programmes being accessible to farmers and a favourable balance between the opportunity cost of selecting for low emissions (which depends on correlations with other desirable traits) and policy incentives for reduced emissions. In countries using artificial insemination, relatively few bulls sire the majority of the national dairy herd, offering potentially high efficacy of this approach.

### (d) Methane inhibitors: seaweed

Algae of the genus *Asparagopsis* have been shown to reduce ruminant CH_4_ emissions by 20–98%, although the persistence of this effect over multiple seasons remains unclear [88,89]. The role of bromoform and bromochloromethane as active ingredients in *Asparagopsis* raises challenges from a regulatory and market acceptability perspective, given that both substances are confirmed animal carcinogens and probable/possible human carcinogens. Animal trials have detected residues in urine and milk [90], but no detrimental effects on meat quality [91]. There are also open questions regarding palatability to livestock, animal health and the ability to produce and supply seaweed at a large scale, especially to extensively grazed livestock [88–90,92].

However, if these concerns can be addressed, a CH_4_-inhibiting seaweed feed additive could be commercially available within the next few years. Additionally, if the inclusion of bromoform in animal feed gains market and regulatory acceptance, this would open the opportunity to produce bromoform cheaply via commercial chemical processes and provide it to animals directly and more consistently (e.g. via a bolus or other feed supplements) rather than through seaweed.

Table 1 summarizes our assessment of these key novel technologies, their current status and applicability, and confidence in future applicability. Collectively, these selected interventions could add considerably to the supply-side mitigation potential identified in sectoral studies and increase the feasibility of achieving deep reductions in CH_4_ emissions from livestock systems by 2050 and beyond. We argue that such options should be included, at least as a sensitivity test, in marginal abatement cost curves and long-term mitigation pathways of IAMs [94,95]. Widespread application of inhibitors and low-emissions breeding is no more speculative by 2050 than the deployment of BECCS at the scale of multiple gigatons per year, which is a routine component of many IAMs [7]. Reliance on speculative technologies to achieve long-term mitigation goals poses clear risks and is contested [96–98], but we suggest that excluding technologies that are already in the process of commercialization presents a skewed picture, not least because their exclusion hides the global benefit of policies to support their further development, commercialization and adoption.

**Table 1.** Key emerging mitigation technologies for livestock CH_4_, their applicability and key constraints across systems, relative emissions reduction, estimated global mitigation potential including constraints on adoption, and timing and confidence in commercial availability. Relative reductions and mitigation potentials are based on expert judgement given the developments set out in the main text and assume continued research, development and commercialization, subject to regulatory and market approval. See notes for details.

technology	applicability	key constraint	relative emissions reduction	mitigation potential in 2050^a^ (Mt CH_4_ yr^−1^)	widespread commercial availability (confidence)^b^
CH_4_ inhibitors	TMR systems	cost	30%	0.8	2025 (high confidence)
	intensive grazing systems (bolus, slow release)	cost	20–30%	5–8	2030 (medium confidence)
CH_4_ vaccine	most systems	sustained R&D, veterinary services, cost	30% (assumed, pending proof of concept)	11–28	2050 (medium confidence)
low-emissions breeding	most systems	breeding programme	1% per year,15% maximum	2–9	sheep: 2030 (high confidence) cattle: 2035 (medium confidence)
seaweed	TMR systems	global-scale production, cost, toxicology, regulatory and market acceptance	20–50%	0.5–1	2030 (insufficient evidence for confidence level)
	possibly intensive grazing systems			1–10	

^a^Global mitigation potentials depend critically on adoption rates including policy incentives; numbers illustrate orders of magnitude, not predictions. We used global modelled emissions for different livestock systems in the year 2010 by GLEAM [93] and increased those emissions by 30% to approximate baseline emissions in 2050. The following adoption rates were used to illustrate global mitigation potentials: *CH_4_ inhibitors:* TMR: 100% adoption in all feedlot systems; Intensive grazing low: 20% efficacy, 50% adoption in mixed systems in high and upper middle income countries; intensive grazing high: 30% efficacy, 50% adoption in mixed systems in high, upper middle and lower middle income countries. *CH_4_ vaccine:* low: 50% adoption in high and upper middle income countries; high: 100% adoption in high and upper middle income countries, 50% adoption in lower-middle income countries. *Low-emissions breeding:* low: 50% adoption in high income countries only; high: 50% adoption in high, upper middle and lower middle income countries. *Seaweed:* TMR: 100% adoption in feedlots with 20% and 50% efficacy; intensive grazing low: 20% efficacy, 50% adoption in high income countries only; Intensive grazing high: 50% efficacy, 50% adoption in high and upper middle income countries.

^b^Confidence reflects expert judgement about availability, given recent progress and agreement in available literature regarding pathways or fundamental barriers to success.

## 4. Integrating methane mitigation into a broader context

While a focus on targeted CH_4_ mitigation is beneficial, especially for technology development, it excludes consideration of the land footprint of livestock and its carbon opportunity cost, and nitrous oxide (N_2_O) emissions. Livestock systems occupy more than 80% of the total agricultural land area, either directly for grazing or indirectly for the production of feed [99]. Freeing some of this land to allow more carbon efficient uses (e.g. afforestation, agroforestry, or production of biofuels; [100]) without compromising food security could achieve significant additional mitigation from agricultural landscapes [9,59,101,102].

Hayek *et al*. [103] estimated the cumulative carbon opportunity cost of animal food production at 332–547 Gt CO_2_ to 2050, based on a global diet with reduced animal-sourced foods as proposed by Willett *et al*. [101] and assuming that land no longer needed to support livestock systems would be used instead to sequester carbon in different ways. This study, while ambitious in its assumptions, suggests that the carbon opportunity cost of livestock systems could be significantly greater than the impact on the remaining carbon budget of approximately 136 Gt CO_2_ from actions to mitigate livestock CH_4_ emissions quantified in our study.

Integrated consideration of emissions and sequestration potentials across agricultural landscapes underlines the importance of demand-side responses. Reducing demand for livestock not only complements supply-side interventions to reduce direct emissions and ensure that intensification is sustainable, but also opens new choices for the use of finite land resources. Sequestering carbon from the atmosphere at rates necessary to help achieve net-zero CO_2_ emissions globally within the next few decades will require additional land. This land can only be spared, without compromising food security, if global demand shifts towards not only less emissions-intensive but also less land-intensive food production systems, including through sustainable intensification [104,105].

Nonetheless, achieving transformative changes in demand and land use will require grappling with several important challenges, and the wider set of social and environmental roles that livestock systems perform needs to be considered [106].

One consideration is that adequate human nutrition requires more than a simple replacement of the calorific value of livestock with plant products. Nutritionally balanced substitutions, including micronutrients, are not necessarily feasible and affordable for all vulnerable populations, and health outcomes depend strongly on detailed assumptions about diet composition [107–113]. Cultured meat and dairy are relevant emerging substitutions [114,115], but availability and affordability for vulnerable populations remain open questions, and their overall climate impact compared to traditional farming depends on the time horizon considered and energy emissions [116].

A second challenge is that soil carbon stocks under pastures are generally high, and shifts to cropland result in a period of CO_2_ emissions; by contrast, integrating pastures into cropping systems has been identified as one of the most effective soil carbon management strategies (e.g. [117,118]). Lightly grazed and natural grasslands have recently been identified as large CO_2_ sinks in response to rising temperatures, carbon-dioxide fertilization and nitrogen deposition [119], and changes in the management of current pasturelands have well-documented if modest potential to enhance soil carbon stocks in many locations [120–122]. Climate change will also require adaptation of land management practices, and it will be important to integrate mitigation and adaptation strategies [106]. Land-use changes thus need to consider the net balance of emissions over a range of time horizons and climatic constraints.

A third challenge comes from the roughly 1.3 billion human livelihoods currently linked to livestock systems. In large parts of the developing world, mixed crop-livestock systems form the backbone of livelihoods, food production and income generation in predominantly small and medium-sized farms. Such systems produce over 60% of the meat and milk and more than 50% of crops globally [2,123–125]. In many originally forested lands, pastures also provide landscape heterogeneity that supports biodiversity, delivers recreational and heritage values and ensures a diversity of employment and social networks [36,126,127]. Silvopasture, renewable energy generation, farm- and eco-tourism offer land-use opportunities that can coexist with, rather than replace livestock systems and the rural communities dependent on them [128–130].

Adopting a land-use perspective to facilitate more environmentally friendly food production systems could contribute to Sustainable Development Goals and improve governance and policy coherence across multiple land uses and users. By contrast, a sole focus on maximizing the efficiency of land resources for nutrition and carbon sequestration risks overlooking the diverse social and cultural roles of livestock systems. The need to provide for a ‘just transition’ for livestock farmers under ambitious climate policies remains underexplored in academic literature and policy [131–134].

## 5. Conclusion

We use the concept of marginal warming to describe the increase in temperature that would occur with, compared to without, a given set of emissions, which directly relates to the warming that could be avoided through future mitigation actions. Our analysis demonstrates that the marginal warming from future livestock CH_4_ emissions amounts to more than one-third of the marginal warming from CO_2_ emissions in mitigation pathways consistent with limiting warming to 1.5°C. We find that failure to reduce livestock CH_4_ emissions would reduce the remaining carbon budget consistent with this temperature goal by almost one-quarter and would make it substantially less feasible to limit warming to 1.5°C. The effect is less severe for a goal of remaining well below 2°C but still amounts to more than 10% of the remaining carbon budget.

Options to reduce livestock CH_4_ emissions exist both on the supply and demand side, but both face significant challenges in terms of implementation. We review recent progress in novel technologies and find that CH_4_ inhibitors, vaccines, low-emissions breeding and the use of certain types of seaweed as feed additives could potentially significantly increase the supply-side mitigation potential and make deep emission reductions more feasible. We suggest that such technologies should be considered routinely in long-term mitigation scenarios, at least as sensitivity tests, given that most are by now no more speculative than gigatonne deployment of BECCS envisaged in many scenarios. Their potential future contributions should not be used as a reason to delay mitigation in the near term using existing practices, but their inclusion would demonstrate the need for and global benefit of policies to support their further development and commercialization and to spur further research and development.

However, a singular focus on reducing CH_4_ emissions from livestock is problematic, given the multiple roles that livestock play in diverse landscapes. Not only are livestock a source of other emissions (N_2_O and CO_2_ from land clearing), but their large land footprint also constitutes a significant carbon opportunity cost. Measures to reduce demand for emissions-intensive livestock products through dietary change and reduced food loss and waste are essential to not only allow emission reductions but also additional carbon sequestration without threatening food security.

Nuanced policies and transitions will be needed to manage trade-offs relating to soil carbon, biodiversity and wider ecosystem services. More fundamentally, the livelihoods of more than one billion people are supported by current livestock systems, and policies aimed to reduce demand for livestock systems will need to provide for a ‘just transition’ for those livelihoods. However, the literature dealing with producer perspectives and offering pathways for a gradual transition is distinctly underdeveloped, and we suggest that significant efforts are needed to ensure that mitigation pathways consistent with 1.5°C or well below 2°C do not create new or exacerbate existing inequalities and vulnerabilities.

Most fundamentally though, none of the mitigation pathways and options discussed in our study will come to pass without targeted policies to address greenhouse gas emissions, reduce the global demand for emissions-intensive livestock products and provide for transitions of those most affected by the necessary transformative changes. The significant potential for the reduction of livestock CH_4_ emissions can only be realized if agriculture, and livestock systems in particular, become part of mainstream climate policies, while recognizing their unique and multiple interacting social, cultural and environmental functions.

## Footnotes

1 The Paris Agreement aims to strengthen the global response to climate change, including by ‘holding the increase in the global average temperature to well below 2°C above pre-industrial levels and pursuing efforts to limit the temperature increase to 1.5°C above pre-industrial levels’ (Article 2.1(a)).

## Data accessibility

The paper uses only publicly available datasets from the IPCC database supporting the Special Report on Global Warming of 1.5° (Huppmann *et al.*, doi:10.5281/ZENODO.3363345) [42] and the publicly accessible simple climate model MAGICC (http://live.magicc.org/). As such and based on advice from the editor, no separate upload of data is required.
